# Preparation of Macrobicyclic Cryptands for Radiometal Complexation: Preparation of Macropa‐Based Cryptands for Efficient Complexation of Radiometals

**DOI:** 10.1002/jlcr.4136

**Published:** 2025-03-05

**Authors:** Laura Höffmann, Magdalena Blei, Falco Reissig, Klaus Kopka, Constantin Mamat

**Affiliations:** ^1^ Helmholtz‐Zentrum Dresden‐Rossendorf Institut für Radiopharmazeutische Krebsforschung Dresden Germany; ^2^ Technische Universität Dresden Fakultät Chemie und Lebensmittelchemie Dresden Germany; ^3^ National Center for Tumor Diseases (NCT) University Cancer Center, University Hospital Carl Gustav Carus Dresden Dresden Germany; ^4^ German Cancer Consortium (DKTK), Partner Site Dresden Dresden Germany

**Keywords:** cryptands, NMR titration, radiolabeling, stability

## Abstract

Macrobicyclic cryptands and especially derivatives with functionalized side arms (picolinate, pyrimidine carboxylate, and bipyridine carboxylate) are able to complex metal ions effectively. In this regard, four new functionalized cryptands were prepared in a convenient two‐step synthesis procedure starting from basic compound 4,10,16,22,27‐pentaoxa‐1,7,13,19‐tetraazabicyclo[11.11.5]nonacosane and fully characterized. Their complexation behavior was tested via ^1^H NMR titration with Ba^2+^, Sc^3+^, La^3+^, Lu^3+^, In^3+^, and Pb^2+^ pointing out log *K* values between 1.4 and 4.0. Radiolabeling with selected cations of radiopharmaceutical relevance (^131^Ba, ^225^Ac, and ^133^La) was performed.

## Graphical abstract

Four macrobicyclic cryptands with pendant side arms were synthesized und their ability to coordinate (radio)metals of interest was tested by ^1^H NMR and radiolabeling.

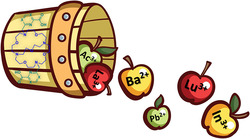

## 1 Introduction

**FIGURE 1:**
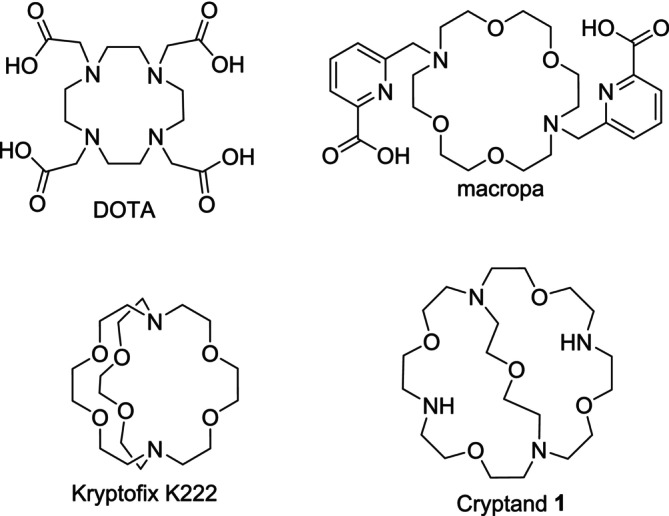
Commonly used ligand DOTA and macropa for radiopharmaceutical applications as well as cryptand Kryptofix K222 especially for the complexation of heavy alkaline earth metal ions and basic cryptand **1** used in this paper.

Molecular baskets such as cryptands based on functionalized macrobicyclic polyethers are known to be effective ligands for alkaline and alkaline earth metal ions [1]. Kryptofix K222 also known as 4,7,13,16,21,24‐hexaoxa‐1,10‐diazabicyclo[8.8.8]hexacosan with 2.8 Å cavity size [2] is the most prominent representative forming complexes with Ba^2+^ > Sr^2+^ > K^+^ > Ca^2+^ > Na^+^ > Li^+^, Mg^2+^ [3]. Cryptands with other cavity size and with additional functional groups also display complexation abilities towards these cations. On the other hand, DOTA—the gold standard in nuclear medicine—displays favorable properties for the complexation of many (radio)metal cations [4] such as In^3+^, Lu^3+^, Tb^3+^, Ac^3+^, or Sc^3+^ [5, 6]. Additionally, macropa has complexation advantages over DOTA with certain cations like Ac^3+^, Pb^2+^, or La^3+^ [7, 8, 9]. Furthermore, the complexes with the highest stability were formed with Ba^2+^ [10, 11, 12] and Ra^2+^ [13, 14] so far. That makes the combination of a molecular basket consisting of cryptands and the functional groups coming from the macrocycles advantageous for the development of new chelating agents. The structures of DOTA, macropa, and K222 are displayed in Figure 1.

The stability of the complexes plays a central role for the development of radiometal complexes applied in radiopharmacy and nuclear medicine [15]. To raise the stability of metal complexes in general, the molecular structure of the ligand (open‐chain, macrocycle, or cryptand), the type and the number of the ligand donor atoms as well as the nature of metal ion has to be considered [16]. Additionally, solvent molecules or biomolecules of the biological system can compete as well with the donor groups of the ligands for the coordination sites of the central cation. Thus, the determination of thermodynamic parameters has still been the subject of extensive research [17]. In this paper, we describe the synthesis of new functional side arms for the modification of the cryptand skeleton as well as the complexation monitored by NMR and radiolabeling of four new functionalized cryptands with cations of Ba, Sc, La, Lu, In, and Pb.

## 2 Results and Discussion

Cryptands have been described, among others, as effective ligands for heavy group 2 metal cations [18]. Cryptand **1** has been used in the past as basis for the preparation of macrotricyclic cryptands [19, 20] suitable for the complexation of group 1 metal cations and anions [21]. For this reason, we have chosen cryptand **1** as molecular skeleton and starting point for our new chelating system containing additional donor functions. Both two secondary amine functions of **1** allow the convenient introduction of additional side arms for the later complexation of selected radiometal cations. For this purpose, the picolinate moiety of macropa seems to be favorable. To adjust the electronic and steric properties, new aromatic side arms based on diazine and bipyridine have been discussed for this purpose.

### 2.1 Synthesis of the Aromatic Side Arms

**FIGURE 2:**
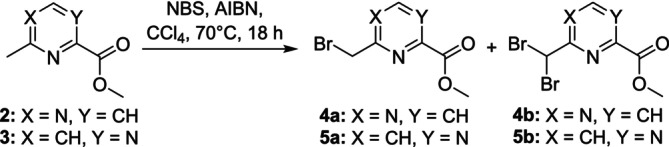
Synthesis of the brominated side arms **4a** and **5a** and dibrominated by‐products **4b** and **5b** using radical substitution mechanism.

To furnish cryptand **1** with side arms containing additional donor functions, three new side arms were prepared in addition to the pyridine side arm used in the macropa chelator. For this purpose, two regioisomeric methylpyrimidine carboxylic esters **2** and **3** were brominated at the benzylic methyl group under radical conditions using *N*‐bromo succinimide and AIBN as initiator to trigger the radical reaction (Figure 2). Both dibrominated by‐products **4b** and **5b** were obtained in a small proportion in addition to the desired mono‐brominated compounds **4a** (43%) and **5a** (20%).

**FIGURE 3:**
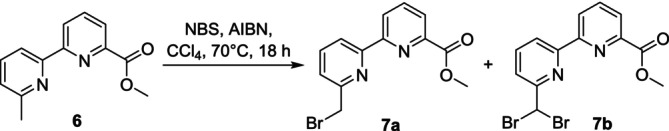
Synthesis of the brominated side arm **7a** and dibrominated by‐products **7b** using the radical substitution reaction.

Methyl 6′‐methyl‐[2,2′‐bipyridine]‐6‐carboxylate (**6**) [22], a moiety with a high steric demand and with an additional aromatic donor function, was also foreseen for a use as side arm and was therefore brominated [23] to achieve the possibility for a connection with cryptand **1**. Here, again, the dibrominated by‐product **7b** was obtained in addition to the desired mono‐brominated compound **7a** (Figure 3). Both compounds were separated by automated flash‐chromatography to yield **7a** in 55% and the by‐product **7b** in a minor content.

### 2.2 Cryptand Functionalization With Side Arms

**FIGURE 4:**
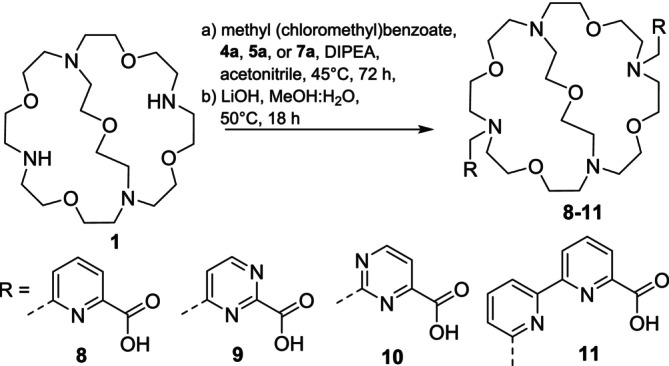
Synthesis of the modified cryptands **8**–**11** in a two‐step synthesis procedure starting from cryptand **1**.

All four mono‐brominated side arms (picolinate, 2 pyrimidine carboxylates, and bipyridyl carboxylate) were used for the functionalization of the cryptand backbone **1**. The picolinate moiety [24] was used in comparison with the macropa ligand, both pyrimidine carboxylates to increase the electron withdrawing effect and bipyridyl carboxylate to raise the coordination sites from 13 to 15 donors and to raise the steric demand. At first, two equivalents of methyl 6‐(chloromethyl)picolinate were reacted with one equivalent of cryptand **1** under basic conditions with DIPEA in acetonitrile to yield the diester of modified cryptand **8** (yield 68%), which was saponified using LiOH in a 1:1 mixture of water and methanol to yield the final cryptand **8** in 25% yield over 2 steps. Afterwards, the side arms **4a**, **5a**, and **7a** were reacted with **1** with DIPEA as base in acetonitrile yielding the methyl esters of **9** (39%), **10** (65%), and **11** (76%), followed by the saponification step of all diesters to yield the final desired cryptands **9** (18%), **10** (10%), and **11** (15%), after two steps. The final purification for all cryptands was accomplished using semi‐preparative HPLC. The reactions are shown in Figure 4.

### 2.3 NMR Titrations

One of our main tasks was the stable complexation of Ba^2+^ using the developed cryptands, because ^131^Ba is a surrogate for ^223^Ra and ^224^Ra [11]. To evaluate the complexation potential of the resulting cryptands **8**–**11**, other radiopharmaceutically relevant radiometals were additionally focused such as ^133^La, ^43/44/47^Sc, ^111^In, ^177^Lu, and ^203/212^Pb. For this purpose, nonradioactive isotopes of Ba, La, Sc, Pb, Lu, and In were used for ^1^H NMR titrations as we did that in the past with other ligand systems [25]. To reach the aim, the ligands **8**–**11** were dissolved in D_2_O and aliquots of the metal salts were added. The solution was allowed to stand for 10 min between every titration step and the measurement recording. At the end, every sample was heated to 90°C for 30 min to ensure complete complexation.

**FIGURE 5:**
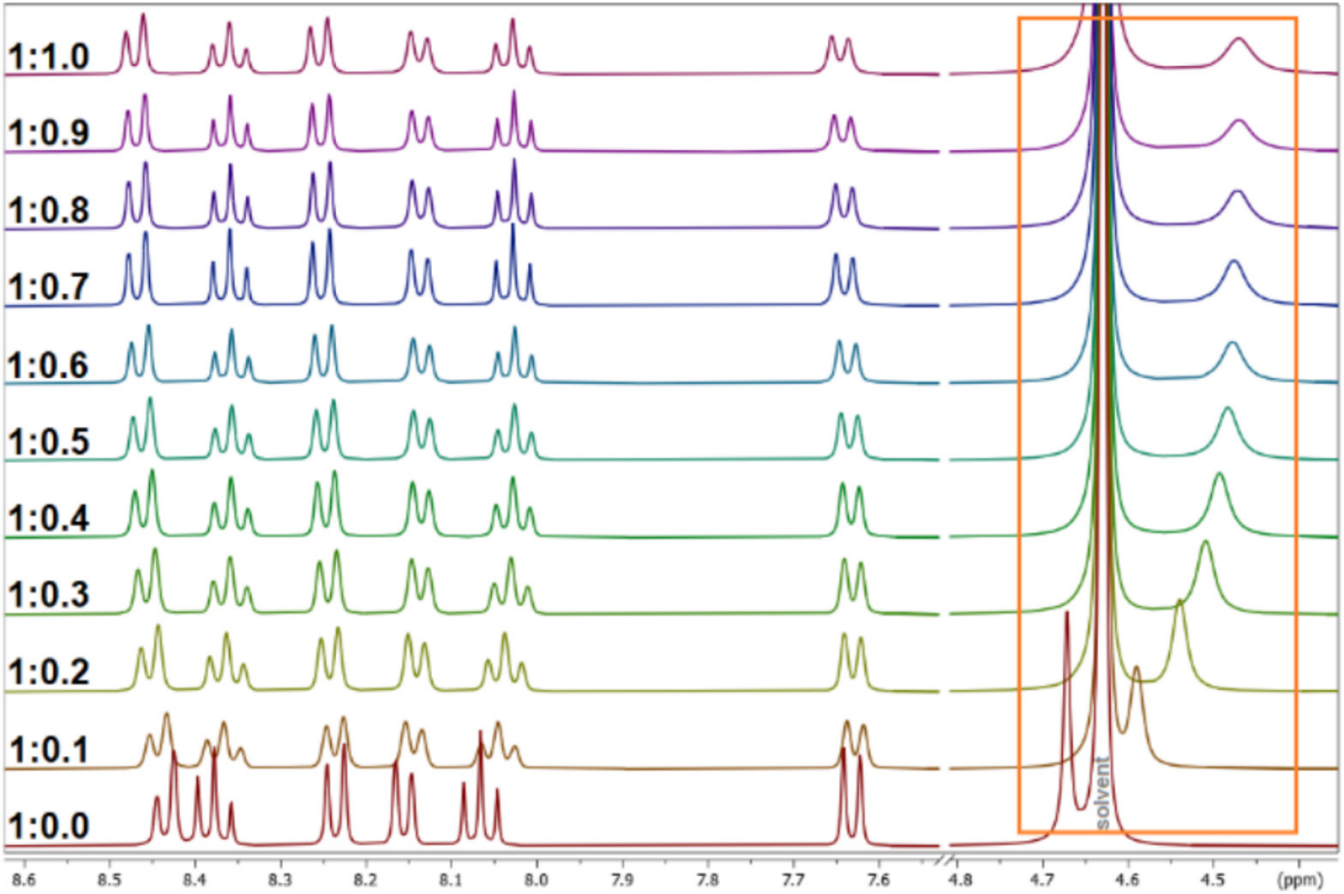
^1^H NMR titration spectra (selected regions of interest) of ligand **11** with different aliquots of Lu^3+^ in D_2_O.

**FIGURE 6:**
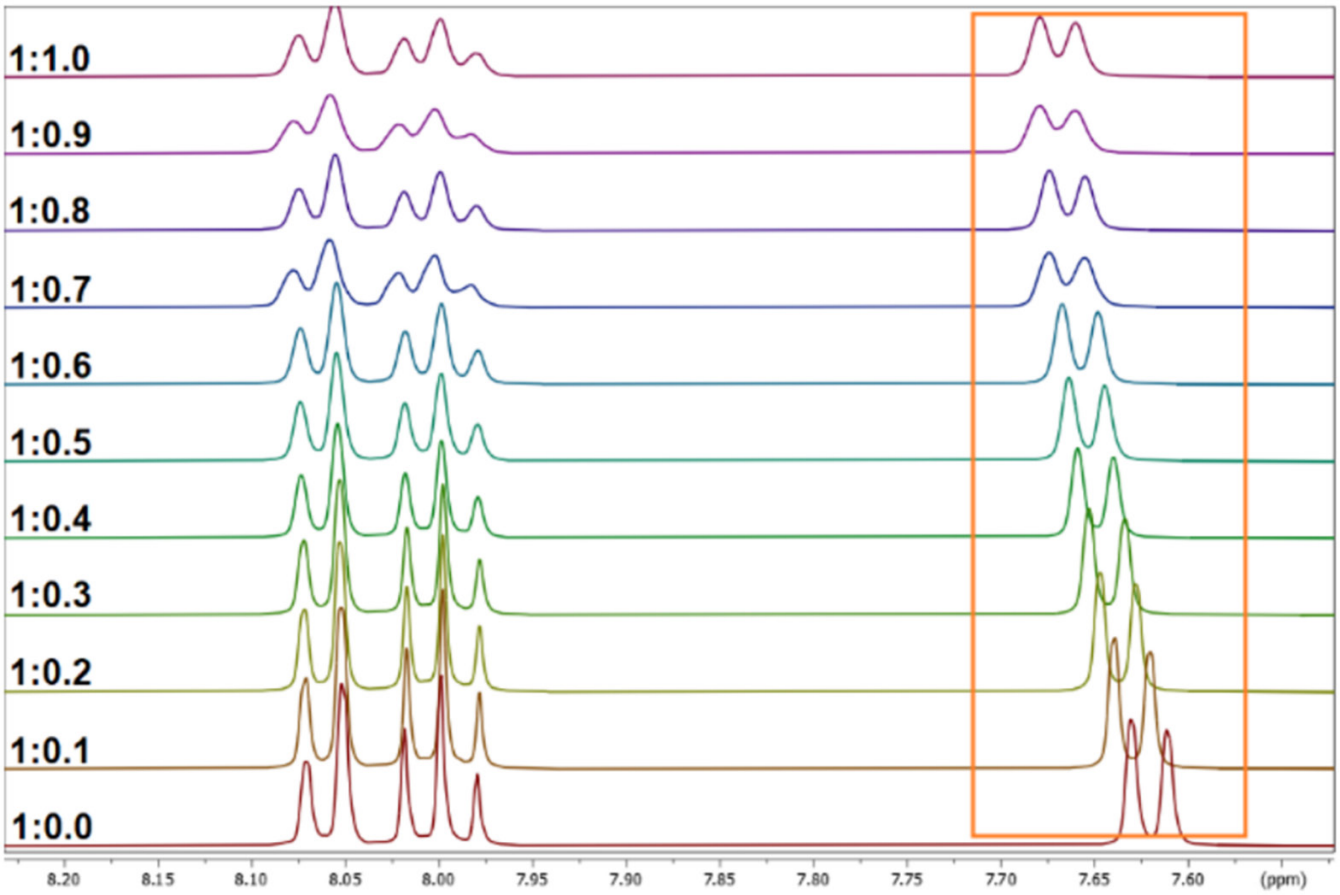
^1^H NMR titration spectra (aromatic region of interest) of ligand **8** with different aliquots of In^3+^ in D_2_O.

As a first result, no change in chemical shifts was observed after the addition of Ba^2+^, La^3+^, Sc^3+^, and Pb^2+^ to all ligands, even after heating. In these cases, no complexation was observed. In the case of Lu^3+^ and In^3+^, the signals in the ^1^H NMR spectra are shifting indicating a complexation via a fast exchange. Examples for an NMR titration of ligand **11** with Lu^3+^ and ligand **8** with In^3+^ are expressed in Figures 5 and 6, respectively. The ^1^H NMR titration spectra of the other ligands with Lu^3+^ and In^3+^ can be found in Data S1.

The change in chemical shifts can be used to determine the complex stability constants (log *K*) with help of the WinEQNMR2 software [26]. It was possible to calculate log *K* values for all cryptands **8**–**11** with Lu^3+^ and for In^3+^. The results are expressed in Table 1. The determined stability constants for In‐ and Lu‐complexes with ligands **8**–**11** are too low compared to the respective values found for In‐DOTA [27] and Lu‐DOTA [28] and therefore far away for a radiopharmaceutical application. Furthermore, a second species was observed during the titration of ligand **10** with In^3+^. In this case, a calculation of the log *K* value was not possible.

**TABLE 1:** Association constants of ligands **8**–**11** with Lu^3+^ and In^3+^.

Ligand	log *K*_as_ (Lu^3+^)	Log *K*_as_ (In^3+^)
**8**	2.2	2.0
**9**	4.0	1.4
**10**	3.4	—
**8**	**11**	2.7
macropa	7.3 [9]	—
DOTA	25.4 [26]	24.5 [27]

### 2.4 Radiolabeling

To further evaluate the potential of the cryptands **8**–**11**, a radiolabeling procedure was elaborated using ^131^Ba, ^133^La, and ^225^Ac. Ligand solutions with concentrations of 10^–3^, 10^–4^, 10^–5^, 10^–6^, and 10^–7^ M were prepared and compared with macropa (c = 10^–3^ M, 10^–4^ M, and 10^–5^ M) as standard. Next, aliquots of activity (exemplarily 100 kBq [^131^Ba]Ba^2+^, [^133^La]La^3+^, and [^225^Ac]Ac^3+^, respectively) were added, and the mixture was shaken at 25°C for 1 h. After labeling, radio‐TLCs analyses were performed with two different systems (normal phase: NP and reverse phase: RP) [9, 11] to monitor the quality of the labeling reaction.

**FIGURE 7:**
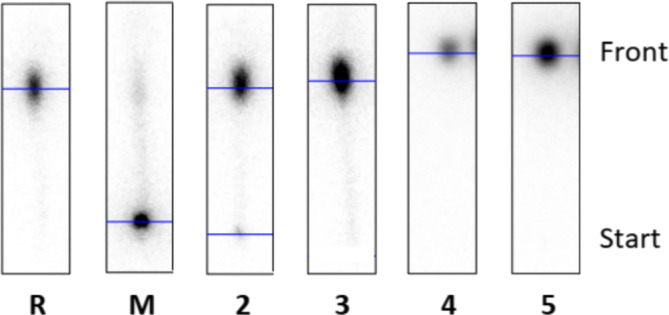
Radio‐TLC's (normal phase: NP) of reference **R** (free [^131^Ba]Ba^2+^), macropa **M** (c = 10^–3^ M), and cryptand **10** at the following concentrations: 10^–2^ M (**2**), 10^–3^ M (**3**), 10^–4^ M (**4**), and 10^–5^ M (**5**) after radiolabeling with barium‐131 (100 kBq).

As a result, none of the present ligands **8**–**11** was appropriate to be fully labeled with [^131^Ba]Ba^2+^ and [^225^Ac]Ac^3+^. Most of the time, the only radioactivity was found at the front using the normal phase system, which implies that [^131^Ba]Ba^2+^ ions were free and Ba‐EDTA complexes formed and move with the front, exemplarily shown in Figure 7 for the labeling trial of cryptand **10** with [^131^Ba]Ba^2+^. To illustrate the difference to a successful complexation, the chromatogram of macropa (M) is also shown in Figure 7, in which the intense [^131^Ba]Ba‐mcp (10^–3^ M) spot can be found at the start of the TLC.

**FIGURE 8:**
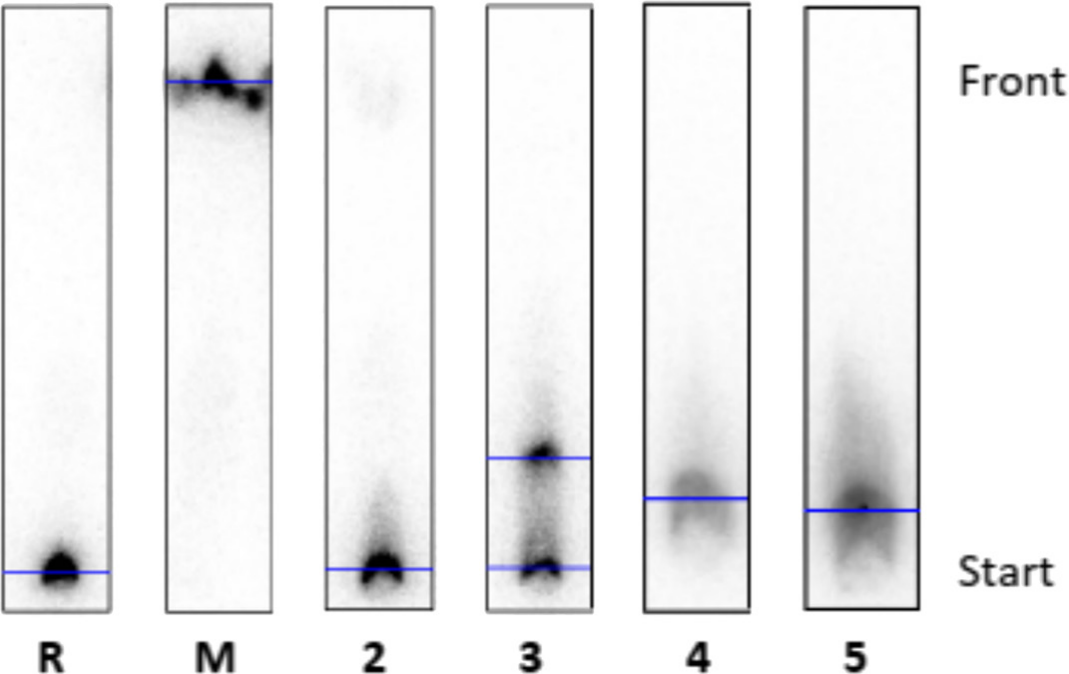
Radio‐TLC's (reverse phase: RP) of reference **R** (free [^131^Ba]Ba^2+^), macropa **M** (c = 10^–3^ M), and cryptand **10** at the following concentrations: 10^–2^ M (**2**), 10^–3^ M (**3**), 10^–4^ M (**4**), and 10^–5^ M (**5**) after radiolabeling with barium‐131 (100 kBq).

When using the RP‐TLC plates, the spots of the free barium ions should be found at the start of the chromatogram. For compound **10** (Figure 8), a second spot was observed, which indicates partial complexation. Integration was used to determine the proportion of free [^131^Ba]Ba^2+^ and the formed [^131^Ba]Ba‐cryptand **10**. The result was 41% bound and 58% free Ba^2+^ at a cryptand concentration of 10^–3^ M. At a ligand concentration of 10^–4^ M, 37% of [^131^Ba]Ba‐cryptand **10** were formed, until finally at a concentration of 10^–5^ M, complexation formation was no longer observed.

For the radiolabeling with actinium‐225 and lanthanum‐133, ligand solutions with concentrations of 10^–5^ M, 10^–6^ M, and 10^–7^ M were used. Of the four cryptands, only cryptand **2** showed very low complexation (max. 1% RCC) at a ligand concentration of 10^–5^ M that may be due to the high excess of EDTA, which acts as a competing ligand and ultimately leads to recomplexation; nevertheless, similar chelators like macropa achieve radiochemical conversions > 99% until 10^–7^ M under the same conditions [8]. Based on the investigations carried out, it was shown that the synthesized cryptands are not suitable as complexing agents for Ac^3+^ and La^3+^ ions, as they prove to be unstable against EDTA with increasing dilution.

## 3 Conclusion

A straightforward synthesis route was elaborated for the preparation of functionalized cryptands based on the K221 skeleton. For this purpose, three aromatic side arms consisting of pyrimidine carboxylate and bipyridyl carboxylate were prepared in addition to the pyridine carboxylate. In the next step, all side arms were reacted with cryptand **1**, delivering the desired functionalized cryptands **8**–**11** after saponification and purification via semi‐preparative HPLC. ^1^H NMR titrations were done with Ba^2+^, Sc^3+^, Pb^2+^, La^3+^, Lu^3+^, and In^3+^ solutions to investigate possible complex formation and to determine an association constant. The titrations with Lu^3+^ and In^3+^ led to a change in chemical shifts in the ^1^H NMR spectrum, because only weak or no changes in chemical shifts were observed for the other metal ions indicating no complex formation. As a result of these calculations, log *K*_a_ between 1.3 and 4.0 were obtained, which conclude that the cryptands are not capable of stable complexation with Lu^3+^ and In^3+^. As the number of coordination sites, of which there should be a sufficient number, and the size of the intramolecular cavity also play a role, it can be assumed that the cryptands are too large for the investigated ions, and therefore, no stable complex can be formed. Finally, radiolabeling with [^131^Ba]Ba^2+^, [^133^La]La^3+^, and [^225^Ac]Ac^3+^ were accomplished, pointing out that all cryptands are not suitable for radiopharmaceutical applications with the tested radiometals.

## 4 Materials and Methods

### 4.1 General

All chemicals used in this work were purchased from the manufacturers and used without further purification for the corresponding syntheses. Anhydrous solvents were obtained commercially from ACROS, TCI, BLD Pharm or Sigma‐Aldrich and used without further purification. Aqueous solutions were prepared using double deionized, sterile filtered water (Merck Millipore purification system). For normal‐phase (NP) thin‐layer chromatography, aluminum plates from Merck with silica gel 60 and the fluorescent indicator F_254_ were used as stationary phase. The substance to be analyzed was applied in the dissolved state, and the chromatogram was developed in the specified running medium mixture. The evaluation was carried out by UV detection (254 and 366 nm). The NMR spectra used for structure elucidation were recorded on two devices from AGILENT with the device designations DD2‐600 MHz Spectrometer and DD2‐400 MHz Spectrometer with ProbeOne probes. The samples were dissolved in deuterated solvents from DEUTERO and measured in 5 mm glass tubes at 25°C. The frequencies at which the respective nuclei were measured are DD2‐400 MHz: ^1^H (400 MHz), ^13^C (101 MHz) and DD2‐600 MHz: ^1^H (600 MHz), ^13^C (151 MHz). MestreNova was used to evaluate the spectra. All ^1^H and ^13^C NMR spectra were calibrated to the signals of the non‐deuterated solvent components. For each signal, the respective chemical shift δ in ppm, the coupling constant *J* in Hertz, the number of associated nuclei and the assignment to the respective structural element are given. The mass spectra were measured on the expression CMS device from ADVION. Positive ions were detected. The mass‐to‐charge ratio (*m/z*) and the assignment of the signals are indicated.

The purification of the corresponding compounds was carried out using automatic flash column chromatography (normal phase) on Isolera Four or Select from Biotage. Pre‐packed separation columns (Biotage Sfär Silica HC D) of various capacities (5 g cartridge up to 1000 mg crude product, 10 g cartridge up to 2000 mg crude product, and 25 g cartridge up to 5 g crude product) were used as the stationary phase. The separation was monitored using a UV detector at 254 and 280 nm, and the fractions were collected accordingly.

The final structures were separated using semi‐preparative HPLC on an Azura system from KNAUER. An Agilent Zorbax 300 SBC18‐25 mm × 12.5 mm with a flow rate of 6 mL/min was used as the separation column. Water and acetonitrile (each with 0.1% TFA) were used as solvents. The product fractions were lyophilized immediately after elution. The separation conditions used are given in the synthesis instructions.

### 4.2 Chemistry

Cryptand **1** was synthesized according to Lehn et al. [19, 20], and the synthesis steps are found in Data S1. Compound **6** was synthesized in three steps according to the literature [29]. The synthesis steps are found in Data S1.

#### 4.2.1 Methyl 2‐(Bromomethyl)pyrimidine‐4‐carboxylate (**4a**)

Methyl 2‐methylpyrimidine‐4‐carboxylate (**2**, 185 mg, 1.22 mmol, 1 eq), *N*‐bromo succinimide (217 mg, 1.22 mmol, 1 eq) and AIBN (37 mg, 0.2 mmol, 0.16 eq) were dissolved in CCl_4_ (4 mL) and stirred at 70°C for 18 h. After TLC control, the mixture was cooled to rt, the solvent was removed, and the crude product was purified by column chromatography (SNAP 25; flow rate: 60 mL/min; CHCl_3_:EtOAc, 0%–20% in 20 CV) to obtain compound **4a** (63 mg, 43%, 0.3 mmol) as a yellowish oil (conversion 50%). *R*_*f*_ = 0.54 (CHCl_3_:EtOAc = 1:1); ^1^H NMR (400 MHz, CDCl_3_): δ = 9.00 (d, ^3^ *J* = 5.0 Hz, 1H, H_Ar_), 7.90 (d, ^3^ *J* = 5.0 Hz, 1H, H_Ar_), 4.71 (s, 2H, CH_2_), 4.04 (s, 3H, CH_3_) ppm; ^13^C NMR (101 MHz, CDCl_3_): δ = 167.3 (CH_Ar_), 164.4 (C=O), 160.2 (C_Ar_), 155.6 (CH_Ar_), 119.5 (CH_Ar_), 53.7 (CH_2_), 33.2 (CH_3_) ppm; MS (ESI+): *m/z* = 231 [M + H]^+^ (^79^Br), 233 [M + H]^+^ (^81^Br).

#### 4.2.2 Methyl 4‐(Bromomethyl)pyrimidine‐2‐carboxylate (**5a**)

Methyl 4‐methylpyrimidine‐2‐carboxylate (**2**, 1.00 g, 6.57 mmol, 1 eq), *N*‐bromo succinimide (1.07 g, 6.01 mmol, 0.91 eq) and AIBN (182 mg, 1.11 mmol, 0.17 eq) were dissolved in CCl_4_ (20 mL) and stirred at 70°C for 18 h. After TLC control, the mixture was cooled to rt, the solvent was removed, and the crude product was purified by column chromatography (SNAP 50; flow rate: 60 mL/min; CHCl_3_:EtOAc, 0%–15% in 20 CV) to obtain compound **5a** (205 mg, 20%, 0.3 mmol) as a yellowish oil (conversion 67%). *R*_*f*_ = 0.54 (CHCl_3_:EtOAc = 1:1); ^1^H NMR (400 MHz, CDCl_3_): δ = 8.94 (d, ^3^ *J* = 5.1 Hz, 1H, H_Ar_), 7.69 (d, ^3^ *J* = 5.1 Hz, 1H, H_Ar_), 4.57 (s, 2H, CH_2_), 4.08 (s, 3H, CH_3_) ppm; ^13^C NMR (101 MHz, CDCl_3_): δ = 166.7 (C=O), 163.7 (C_Ar_), 158.9 (CH_Ar_), 156.4 (C_Ar_), 122.6 (CH_Ar_), 53.9 (CH_2_), 31.2 (CH_3_) ppm; MS (ESI+): *m/z* = 231 [M + H]^+^ (^79^Br), 233 [M + H]^+^ (^81^Br).

#### 4.2.3 Methyl 6′‐(Bromomethyl)‐(2,2′‐bipyridine)‐6‐carboxylate (**7a**)

Methyl 6′‐methyl‐[2,2′‐bipyridine]‐6‐carboxylate (**6**, 123 mg, 0.5 mmol, 1 eq), *N*‐bromo succinimide (96 mg, 0.5 mmol, 1 eq), and AIBN (18 mg, 0.1 mmol, 0.2 eq) were dissolved in CCl_4_ (6 mL) and stirred at 70°C for 20 h. After TLC control, the mixture was cooled to rt, the solvent was removed, and the crude product was purified via automated column chromatography (SNAP 10 cartridge, flow rate 25 mL/min; CHCl_3_:EtOAc + 1% NEt_3_, 0%–15% in 20 CV) to obtain compound **7a** (58 mg, 56%) as a colorless solid (conversion 55%). *R*_*f*_ = 0.65 (CHCl_3_:EtOAc + 0.1% Et_3_N = 9:1), ^1^H NMR (400 MHz, CDCl_3_): δ = 8.67 (d, ^3^ *J* = 7.8 Hz, 1H, H_Ar_), 8.47 (d, ^3^ *J* = 7.8 Hz, 1H, H_Ar_), 8.14 (d, ^3^ *J* = 7.8 Hz, 1H, H_Ar_), 7.97 (t, ^3^ *J* = 7.8 Hz, 1H, H_Ar_), 7.86 (t, ^3^ *J* = 7.8 Hz, 1H, H_Ar_), 7.50 (d, ^3^ *J* = 7.8 Hz, 1H, H_Ar_), 4.64 (s, 2H, CH_2_), 4.04 (s, 3H, CH_3_) ppm; ^13^C NMR: (101 MHz, CDCl_3_) δ = 165.9 (C=O), 158.4 (C_Ar_), 155.7 (C_Ar_), 154.0 (C_Ar_), 147.8 (C_Ar_), 138.3 (CH_Ar_), 138.1 (CH_Ar_), 125.4 (CH_Ar_), 124.4 (CH_Ar_), 121.3 (CH_Ar_), 120.7 (CH_Ar_), 64.0 (CH_3_), 53.0 (CH_2_) ppm; MS (ESI+): *m/z* = 307 [M + H]^+^ (^79^Br), 309 [M + H]^+^ (^81^Br).

#### 4.2.4 6,6′‐((4,10,16,22,27‐Pentaoxa‐1,7,13,19‐tetraazabicyclo[11.11.5]nonacosane‐7,19‐diyl)bis(methylene))dipicolinic Acid (**8**)

Cryptand **1** (211 mg, 0.5 mmol, 1 eq), methyl 6‐(chloromethyl)picolinate (225 mg, 1.3 mmol, 2.6 eq), and DIPEA (214 μL, 1.23 mmol, 2.5 eq) were dissolved in anhydrous acetonitrile (10 mL). The reaction mixture was stirred at 45°C for 72 h. After cooling to rt, the solvent was removed, and the crude product was purified by column chromatography (SNAP 10; flow rate 30 mL/min; EtOH:EtOAc + 1% NEt_3_ 0%–40% in 25 CV). The diester was collected as yellowish solid in a yield of 68% (247 mg, 0.34 mmol). *R*_*f*_ = 0.4 (EtOH:EtOAc + 1% NEt_3_ = 3:2), ^1^H NMR (400 MHz, CDCl_3_): δ = 7.94 (dd, ^3^ *J* = 7.3 Hz, ^4^ *J* = 1.5 Hz, 2H, H_Ar_), 7.77 (t, ^3^ *J* = 7.3 Hz, 2H, H_Ar_), 7.74 (dd, ^3^ *J* = 7.3 Hz, ^4^ *J* = 1.5 Hz, 2H, H_Ar_), 3.92 (s, 6H, CH_3_), 3.89 (s, 4H, CH_2_), 3.77–3.70 (m, 4H, CH_2_), 3.66 (t, ^3^ *J* = 5.0 Hz, 8H, CH_2_), 3.53 (t, ^3^ *J* = 5.0 Hz, 8H, CH_2_), 3.16–3.10 (m, 12H, CH_2_), 2.78 (t, ^3^ *J* = 5.0 Hz, 8H, CH_2_) ppm; ^13^C NMR (101 MHz, CDCl_3_): δ = 165.8 (C=O), 160.2 (CH_Ar_—), 147.3 (C_Ar_—), 137.4 (C—H_Ar_), 126.4 (C—H_Ar_), 123.7 (C—H_Ar_), 69.4 (OCH_2_), 60.8 (NCH_2_), 54.6 (NCH_2_), 54.5 (NCH_2_), 52.9 (CH_3_) ppm; MS (ESI+): *m/z* = 717 [M + H]^+^. Afterwards, the obtained diester (220 mg, 0.3 mmol, 1 eq) and LiOH (23 mg, 0.9 mmol, 3 eq) were dissolved in 12 mL of a 1:1 mixture of deionized water and methanol, and the resulting solution was stirred at 50°C for 18 h. Next, the solvent was removed, and the residue was dissolved in 4 mL of deionized water and filtered through a syringe filter. Semipreparative HPLC (flow rate 6 mL/min; H_2_O + 0.1% TFA:ACN + 0.1% TFA 90:10 → 60:40) was used for purification followed by lyophilization. Compound **8** was obtained as a colorless solid in a yield of 37% (175 mg, 0.11 mmol). *R*_*f*_ = 0.03 (EtOH:EtOAc + 1% NEt_3_ = 3:2); ^1^H NMR (400 MHz, D_2_O): δ = 8.23 (d, ^3^ *J* = 7.8 Hz, 2H, H_Ar_), 8.13 (t, ^3^ *J* = 7.8 Hz, 2H, H_Ar_), 7.77 (d, ^3^ *J* = 7.8 Hz, 2H, H_Ar_), 4.22–3.35 (m, 44H, CH_2_) ppm; ^13^C NMR (101 MHz, D_2_O): δ = 166.9 (C=O), 149.3 (CH_Ar_), 146.8 (C_Ar_), 140.0 (CH_Ar_), 127.7 (C_Ar_), 125.9 (CH_Ar_), 64.6, 64.2, 63.8, 63.3, 57.5, 54.2, 54.0, 53.4, 52.3 (NCH_2_ + OCH_2_) ppm; MS (ESI+): *m/z* = 689 [M + H]^+^.

#### 4.2.5 4,4'‐((4,10,16,22,27‐Pentaoxa‐1,7,13,19‐tetraazabicyclo[11.11.5]nonacosan‐7,19‐diyl)bis(methylene))bis(pyrimidine‐2‐carboxylic Acid) (**9**)

Cryptand **1** (45 mg, 0.11 mmol, 1 eq), compound **5a** (50 mg, 0.22 mmol, 2 eq), and DIPEA (46 μL, 0.26, 2.4 eq) were dissolved in anhydrous acetonitrile (10 mL), and the reaction mixture was stirred at 45°C for 72 h. After TLC control, the solvent was removed, and the crude product was purified by automated column chromatography (SNAP 10; flow rate: 30 mL/min; EtOAc:EtOH + NEt_3_, 0%–50% in 20 CV). The diester was obtained as a light‐yellow solid in a yield of 39% (31 mg, 0.045 mmol). *R*_*f*_ = 0 (EtOAc:EtOH + 1% NEt_3_ = 3:2); ^1^H NMR (400 MHz, CDCl_3_): δ = 8.85 (d, ^3^ *J* = 5.1 Hz, 2H, H_Ar_), 7.93 (d, ^3^ *J* = 5.1 Hz, 2H, H_Ar_), 4.04 (s, 6H, CH_3_), 3.94 (s, 4H, CH_2_), 3.72–3.39 (m, 20H, CH_2_), 2.93–2.67 (m, 20H, CH_2_) ppm; ^13^C NMR (101 MHz, CDCl_3_): δ = 171.8 (C_Ar_), 163.9 (C=O), 157.9 (CH_Ar_), 156.3 (C_Ar_), 121.9 (CH_Ar_), 70.0 (OCH_2_), 69.8 (OCH_2_), 62.9 (NCH_2_), 55.5 (NCH_2_), 55.0 (NCH_2_), 53.7 (CH_3_) ppm. MS (ESI+): *m/z* = 360 [M + H]^2+^. Afterwards, the diester (31 mg, 0.043 mmol, 1 eq) and LiOH (3 mg, 0.13 mmol, 3 eq) were dissolved in 10 mL of a 1:1 mixture of deionized water and methanol, and the resulting solution was stirred at 50°C for 18 h. Next, the solvent was removed, and the residue was dissolved in 400 μL of deionized water and centrifuged. Semipreparative HPLC (flow rate 6 mL/min; H_2_O + 0.1% TFA:ACN + 0.1% TFA 90:10 → 60:40) was used for purification followed by lyophilization. The product **9** was obtained as a colorless solid in a yield of 47% (29 mg, 0.02 mmol). ^1^H NMR (400 MHz, D_2_O): δ = 8.98 (d, ^3^ *J* = 5.2 Hz, 2H, H_Ar_), 7.76 (d, ^3^ *J* = 5.2 Hz, 2H, H_Ar_), 4.90 (s, 4H, CH_2_), 4.25–3.45 (m, 40H, CH_2_) ppm; ^13^C NMR (101 MHz, D_2_O): δ = 168.7 (C=O), 159.9 (C_Ar_), 159.5 (C_Ar_), 158.8 (CH_Ar_), 121.4 (CH_Ar_), 64.4, 63.9, 63.4, 56.5, 54.6, 53.3, 52.5 (NCH_2_ + OCH_2_) ppm; MS (ESI+): *m/z* = 691 [M + H]^+^.

#### 4.2.6 2,2′‐(4,10,16,22,27‐Pentaoxa‐1,7,13,19‐tetraazabicyclo[11.11.5] nonacosan‐7,19‐diyl)bis(methylene))bis(pyrimidine‐4‐carboxylate) (**10**)

Cryptand **1** (150 mg, 0.35 mmol, 1 eq), compound **4a** (166 mg, 0.7 mmol, 2 eq), and DIPEA (152 μL, 0.87 mmol, 2.5 eq) were dissolved in anhydrous acetonitrile (7 mL), and the reaction mixture was stirred at 45 C for 72 h. After TLC control, the solvent was removed, and the crude product was purified by automated column chromatography (SNAP 10; flow rate: 30 mL/min; EtOAc:EtOH + NEt_3_, 0%–50% in 20 CV). The diester was obtained as a brown solid (65%, 167 mg, 0.23 mmol). *R*_*f*_ = 0.18 (EtOAc:EtOH + 1% NEt_3_ = 2:3); ^1^H NMR (400 MHz, CDCl_3_): δ = 8.94 (d, ^3^ *J* = 5.1 Hz, 2H, H_Ar_), 7.26 (d, ^3^ *J* = 5.1 Hz, 2H, H_Ar_), 4.18 (s, 4H, CH_2_), 3.99 (s, 6H, CH_3_), 3.93–3.35 (m, 32H, CH_2_), 3.09–2.88 (m, 8H, CH_2_) ppm; ^13^C NMR (101 MHz, CDCl_3_): δ = 175.7 (C—H_Ar_), 164.5 (C=O), 159.3 (C_Ar_—), 154.9 (C—H_Ar_), 119.2 (C—H_Ar_), 67.5 (OCH_2_), 62.7 (NCH_2_), 58.4 (NCH_2_), 53.5 (NCH_2_), 52.5 (CH_3_) ppm; MS (ESI+): *m/z* = 719 [M + H]^+^. Afterwards, the diester (167 mg, 0.23 mmol, 1 eq) and LiOH (17.4 mg, 0.73 mmol, 3 eq) were dissolved in 12 mL of a 1:1 mixture of deionized water and methanol. The resulting solution was stirred at 50°C for 18 h. Next, the solvent was removed, and the residue was dissolved in 4 mL of deionized water and filtered through a syringe filter and the solvent removed. Semipreparative HPLC (flow rate 6 mL/min; H_2_O + 0.1% TFA:ACN + 0.1% TFA 90:10 → 60:40) was used for purification followed by lyophilization. The product **10** was obtained as a light‐yellow solid (15%, 54.4 mg, 0.034 mmol). ^1^H NMR (400 MHz, D_2_O): δ = 9.13 (d, ^3^ *J* = 5.0 Hz, 2H, H_Ar_), 8.13 (d, ^3^ *J* = 5.0 Hz, 2H, H_Ar_), 4.93 (s, 4H, CH_2_), 4.18–3.49 (m, 40H, CH_2_) ppm; ^13^C NMR (101 MHz, D_2_O): δ = 168.2 (C=O), 160.5 (CH_Ar_), 159.7 (C_Ar_), 159.0 (C_Ar_), 120.2 (CH_Ar_), 64.6, 64.0, 63.5, 57.5, 54.5, 53.3, 52.7 (OCH_2_ + NCH_2_) ppm; MS (ESI+): *m/z* = 691 [M + H]^+^.

#### 4.2.7 6′,6‴‐((4,10,16,22,27‐Pentaoxa‐1,7,13,19‐tetraazabicyclo[11.11.5]nonacosane‐7,19‐diyl)bis(methylene))bis(([2,2′‐bipyridine]‐6‐carboxylic acid)) (**11**)

Cryptand **1** (68 mg, 0.16 mmol, 1 eq), compound **7a** (100 mg, 0.32 mmol, 2 eq), and DIPEA (101 μL, 0.58 mmol, 3.6 eq) were dissolved in anhydrous acetonitrile (10 mL). The reaction mixture was stirred at 45°C for 72 h. After cooling to rt, the solvent was removed, and the product was purified by automated column chromatography (SNAP 10; flow rate: 30 mL/min; EtOAc:EtOH + NEt_3_, 0%–50% in 20 CV). The diester was collected as a light‐yellow solid in a yield of 76% (107 mg, 0.12 mmol). *R*_*f*_ = 0.06 (EtOAc:EtOH + 1% NEt_3_, 3:2); ^1^H NMR (400 MHz, CDCl_3_): δ = 8.60 (d, ^3^ *J* = 7.8 Hz, 2H, H_Ar_), 8.37 (d, ^3^ *J* = 7.7 Hz, 2H, H_Ar_), 8.11 (d, ^3^ *J* = 7.6 Hz, 2H, H_Ar_), 7.93 (t, ^3^ *J* = 7.8 Hz, 2H, H_Ar_), 7.80 (t, ^3^ *J* = 7.8 Hz, 2H, H_Ar_), 7.56 (d, ^3^ *J* = 7.6 Hz, 2H, H_Ar_), 4.02 (s, 6H, CH_3_), 3.93 (s, 4H, CH_2_), 3.63 (m, 20H, CH_2_), 3.03–2.66 (m, 20H, CH_2_) ppm. ^13^C NMR (101 MHz, CDCl_3_): δ = 165.9 (C=O), 156.6 (C_Ar_), 154.6 (C_Ar_), 147.6 (CH_Ar_), 138.0 (CH_Ar_), 137.5 (CH_Ar_), 125.1 (CH_Ar_), 124.4 (CH_Ar_), 124.0 (C_Ar_), 120.1 (C_Ar_), 69.2, 67.7, 67.0, 60.0, 58.6, 55.3, 54.7 (OCH_2_ + NCH_2_), 52.9 (CH_3_) ppm. MS (ESI+): *m/z* = 872 [M + H]^+^. Afterwards, the obtained diester (80 mg, 0.1 mmol, 1 eq) and LiOH (7 mg, 0.3 mmol, 3 eq) were dissolved in 12 mL of a 1:1 mixture of deionized water and methanol, and the resulting solution was stirred at 50°C for 18 h. Next, the solvent was removed, and the residue was dissolved in 400 μL of deionized water and centrifuged. Semipreparative HPLC (flow rate 6 mL/min; H_2_O + 0.1% TFA:ACN + 0.1% TFA 90:10 → 60:40) was used for purification followed by lyophilization. Compound **11** was obtained as a colorless solid in a yield of 20% (35 mg, 0.02 mmol). ^1^H NMR (400 MHz, D_2_O): δ = 8.63 (d, ^3^ *J* = 8.0 Hz, 2H, H_Ar_), 8.57 (t, ^3^ *J* = 7.8 Hz, 2H, H_Ar_), 8.41 (d, ^3^ *J* = 8.0 Hz, 2H, H_Ar_), 8.35 (d, ^3^ *J* = 7.6 Hz, 2H, H_Ar_), 8.23 (t, ^3^ *J* = 7.8 Hz, 2H, H_Ar_), 7.80 (d, ^3^ *J* = 7.7 Hz, 2H, H_Ar_), 4.83 (s, 4H, CH_2_), 4.10 (s, 8H, CH_2_), 3.96 (s, 8H, CH_2_), 3.92 (s, 4H, CH_2_), 3.78 (s, 8H, CH_2_), 3.62–3.55 (m, 12H, CH_2_) ppm; ^13^C NMR (101 MHz, D_2_O): δ = 165.1 (C=O), 149.8 (CH_Ar_), 146.6 (C_Ar_), 145.0 (C_Ar_), 140.4 (CH_Ar_), 127.4 (CH_Ar_), 126.0 (CH_Ar_), 125.6 (CH_Ar_), 123.4 (CH_Ar_), 117.7 (C_Ar_), 114.8 (C_Ar_), 65.0, 64.1, 63.8, 57.0, 53.8, 53.4, 53.1 (OCH_2_ + NCH_2_) ppm. MS (ESI+): *m/z* = 422 [M + 2H]^2+^.

### 4.3 NMR Titration

A solution of the respective ligand **8**–**11** was prepared in D_2_O (20 mg/mL) and 1.0 mL was pipetted in an NMR tube [25]. The spectra of the free ligands were then recorded. All samples were referenced to the residual solvent signal. Then, the complexation of cations with **8**–**11** was studied. A solution of the respective metal salt (Ba(ClO_4_)_2_, LuCl_3_, Pb(ClO_4_)_2_, InCl_3_, La(NO_3_)_3_, or Sc(NO_3_)_3_) was prepared (c_metal_ = 10 × c_ligand_) in the same solvent. Next, stepwise portions (10 μL) of the respective salt solution were added into the NMR tube containing the ligand, and after extensive mixing for 10 and 5 min ultrasound treatment, the complexation‐induced shifts were recorded (standard ^1^H NMR experiment VnmrJ, ns = 8). The addition was continued to a ligand:metal ratio of 1:5 to exclude the formation of a complex with another stoichiometry. The displacements of selected ^1^H NMR signals of ligands **8**–**11** upon addition of the salt solution were used to calculate the complex stability constants. All calculations were performed using the WinEQNMR2 software [24]. The advised range for the data input covers the addition of metal to ligand from 0.1 to 0.9 equivalents. This instruction was followed, and 9 points in this range were measured (steps of 0.1 equiv.) and used for the calculation.

### 4.4 Radiochemistry

Actinium‐225 was purchased as a 0.1 M HCl solution from ITM Munich and used without purification. The production of ^133^La and ^131^Ba was carried out at the TR‐FLEX (ACSI) cyclotron at the HZDR. Lanthanum‐133 (0.05 M HCl) was prepared using the ^134^Ba(p,2n)^133^La nuclear reaction [30]. Barium‐131 (0.1 M HNO_3_) was prepared using the ^133^Cs(p,3n)^131^Ba nuclear reaction [11]. Stock solutions in deionized water and concentrations of 10^–2^ to 10^–6^ M were prepared for the cryptands to be tested. Macropa solutions with concentrations of 10^–2^ to 10^–4^ M were used as reference samples. The reaction approach was selected in such a way that a stock concentration of 10^–2^ M results in a final ligand concentration of 10^–3^ M. The radionuclide solutions of [^131^Ba]Ba^2+^, [^133^La]La^3+^, and [^225^Ac]Ac^3+^ used were diluted accordingly so that 2 μL corresponded to approximately 100 kBq of activity. A 0.2 M ammonium acetate buffer (pH 6) was used as the reaction matrix for the labeling, so that the total volume of 100 μL was reached. The samples were then centrifuged and labeled at 40°C for 1 h. The reaction mixtures were centrifuged again before being applied to the TLC plate.

The TLC plates silica gel 60 F_254_ from MERCK were used as the stationary phase for normal‐phase thin‐layer chromatography. A 2.5 × 10 cm strip was used for each analysis. The application line was drawn at 1 cm. An aqueous 50 mM EDTA solution with pH 7 was used as the mobile phase. Using a pipette, 1 μL of the radiolabel to be examined was applied to the starting line of each TLC plate. The TLC plate was then developed in a TLC chamber with the mobile phase until the running medium had reached three‐fourth of the plate. After drying, the TLC plates were wrapped in Parafilm sealing tape and placed on an imaging plate, which was then read out with a phosphor imager. The radio‐TLCs of the ^225^Ac‐labeling were only analyzed after 24 h. The recorded chromatograms were evaluated using the AIDA 5.1 program.

A cyano‐modified HPTLC silica gel (Nano‐SIL CN, Machery‐Nagel) was used as the stationary phase for reversed‐phase radio thin‐layer chromatography. A 2.5 × 10 cm strip was used for each analysis and the starting line was drawn at 1 cm. A running agent consisting of acetonitrile and water 70:30 with 0.1% TFA was used as the mobile phase. The running agent did not contain any competing complexing agent. Using a pipette, 1 μL of the radiolabeling mixture to be tested was applied to the starting line of each TLC plate. The TLC plate was then developed in a TLC chamber with the mobile phase until the running agent had reached 3/4 of the plate. After drying, the TLC plates were wrapped in Parafilm sealing tape and placed on an imaging plate, which was then read out using a phosphor imager. The recorded chromatograms were evaluated using the AIDA 5.1 program.

## Conflicts of Interest

The authors declare no conflicts of interest.

## Supporting information


**Data S1.** Supporting Information.

## Data Availability

The data that supports the findings of this study are available in the supplementary material of this article.
