# Supplementary material for: Preparation of Macrobicyclic Cryptands for Radiometal Complexation: Preparation of Macropa‐Based Cryptands for Efficient Complexation of Radiometals
Source: J Labelled Comp Radiopharm. 2025 Mar 5;68(3):e4136. doi: 10.1002/jlcr.4136 (PMC11880877; doi:10.1002/jlcr.4136)
Supplement: Supplementary file 1 — Data S1. Supporting Information. [file JLCR-68-0-s001.docx]

**Preparation of Macrobicyclic Cryptands for Radiometal Complexation**

Laura Höffmann^a,b^, Magdalena Blei^a,b^, Falco Reissig^a^, Klaus Kopka^a,b,c,d^, Constantin Mamat^a,b^*

a) Helmholtz-Zentrum Dresden-Rossendorf, Institut für Radiopharmazeutische Krebsforschung, Bautzner Landstr. 400, D‑01328 Dresden, Germany, E-mail: c.mamat@hzdr.de

b) Technische Universität Dresden, Fakultät Chemie und Lebensmittelchemie, D-01062 Dresden, Germany

c) National Center for Tumor Diseases (NCT), University Cancer Center, University Hospital Carl Gustav Carus Dresden, D‑01307 Dresden, Germany

d) German Cancer Consortium (DKTK), Partner Site Dresden, D-01307 Dresden, Germany

**Table of Contents**

Synthesis scheme of cryptand **1** ……………………………………………………………………………………………………….. 2

Synthesis procedures to prepare cryptand **1** ……………………………………………………………………………………. 3

NMR spectra of compounds …………………………………………………………………………………………………………….. 5

NMR Titrations ………………………………………………………………………………………………………………………………. 13

**Synthesis scheme of cryptand 1 according to Lehn et al.**

**Synthesis procedures to prepare cryptand 1**

***N*-Tosyl-2,8-dioxa-5-aza-1,9-nonandicarboxylic acid**

3 g (32 mmol, 2 eq) of chloroacetic acid and 4.09 g (16 mmol, 1 eq) of *N*-tosyl-diethanolamine were dissolved in 80 mL of anhydrous tetrahydrofuran. Subsequently, 2.6 g (0.11 mol, 6.9 eq) sodium hydride (60% in mineral oil) was added in portions. The reaction solution was stirred at 60 °C for 4 d. At the end of the reaction time, the solvent was removed. The residue obtained was dissolved in a little portion of water and washed three times each with 20 mL of petroleum ether and chloroform. The aqueous phase was acidified with 1 M hydrochloric acid to a pH value of 2-3. Then, extracted three times with 20 mL of chloroform. After drying over NaSO_4_, the solvent was removed. The product was obtained as a yellowish oil in a yield of 99 % (5.87 g, 15 mmol). R_f_ = 0.72 (EtOH:EtOAc, 3:2); ^1^H NMR (400 MHz, CDCl_3_): δ = 10.21 (s, 2H, OH), 7.68 (d, ^3^*J* = 8.2 Hz, 2H, ArH), 7.29 (d, ^3^*J* = 8.2 Hz, 2H, ArH), 4.10 (s, 4H, CH_2_), 3.74 (t, ^3^*J* = 5.5 Hz, 4H, CH_2_), 3.38 (t, ^3^*J* = 5.5 Hz, 4H, CH_2_), 2.40 (s, 3H, CH_3_) ppm; ^13^C NMR (101 MHz, CDCl_3_): δ = 174.7 (C=O), 143.8 (C_Ar_), 136.0 (C_Ar_), 129.9 (CH_Ar_), 127.4 (CH_Ar_), 70.7 (CH_2_), 68.0 (OCH_2_), 49.4 (NCH_2_), 21.6 (CH_3_) ppm. MS (ESI(+)): *m/z* = 376 [M+H]^+^ M: 375.39 g/mol

***N*-Tosyl-2,8-dioxa-5-aza-1,9-nonandicarboxylic chloride**

In a 100 mL flask, 2.9 g (7.7 mmol, 1 eq) of *N*-Tosyl-2,8-dioxa-5-aza-1,9-nonandicarboxylic acid was dissolved in 30 mL of tetrachloromethane. Then 3 mL (35 mmol, 4.5 eq) of oxalyl chloride and 0.1 mL of DMF were added as catalyst. The reaction mixture was stirred at 40 °C for 4 hours. Afterwards, the solvent was removed under high vacuum. The product was obtained as a yellowish oil in a yield of 95 % (3.0 g, 7.4 mmol) and subsequently frozen for storage under an argon atmosphere. ^1^H NMR (400 MHz, CDCl_3_): δ = 7.70 (d, ^3^*J* = 8.3 Hz, 2H, ArH), 7.31 (d, ^3^*J* = 8.3 Hz, 2H, ArH), 4.37 (s, 4H, CH_2_), 3.76 (t, ^3^*J* = 5.6 Hz, 4H, CH_2_), 3.43 (t, ^3^*J* = 5.6 Hz, 4H, CH_2_), 2.43 (s, 3H, CH_3_) ppm; ^13^C NMR (101 MHz, CDCl_3_): δ = 171.9 (C=O), 143.8 (C_Ar_), 136.4 (C_Ar_), 129.9 (CH_Ar_), 127.4 (CH_Ar_), 76.2 (CH_2_), 71.1 (OCH_2_), 49.0 (NCH_2_), 21.7 (CH_3_) ppm.

**4.5. 16-Tosyl-1,7,13-trioxa-4,10,16-triazacyclooctadecane-3,11-dione**

2.90 g (7 mmol, 1 eq) of *N*-Tosyl-2,8-dioxa-5-aza-1,9-nonandicarboxylic chloride were dissolved in 30 mL of THF and were transferred in a 30 mL syringe (Ø 2 cm). A solution of 1.49 g (14 mmol, 2 eq) 2,2'-oxybis(ethylamine) was dissolved in 30 mL anhydrous THF and was transferred in a second 30 mL syringe. the two solutions were added to 390 mL of anhydrous THF under argon atmosphere over 7 h using a syringe pump (4.3 mL/h). The reaction solution was then stirred for 72 hours. Afterwards, the resulting precipitate has been removed by filtration, the solvent was removed and the product was purified by column chromatography (SNAP 25 g; flow rate: 25 mL/min; EtOAc:EtOH 0-20% in 20 CV). After removal of the solvent, the product was obtained in a yield of 55 % (1.74 g, 4 mmol) as a light-yellow oil. R_f_ = 0.50 (EtOH: EtOAc, 3:2); ^1^H NMR: (400 MHz, CDCl_3_) δ = 7.69 (d, ^3^*J* = 8.0 Hz, 2H, ArH), 7.32 (d, ^3^*J* = 8.0 Hz, 2H, ArH), 7.09 (t, ^3^*J* = 4.9 Hz, 2H, NH), 3.94 (s, 4H, CH_2_), 3.67 (t, ^3^*J* = 4.9 Hz, 4H, CH_2_), 3.57 (t, ^3^*J* = 4.9 Hz, 4H, CH_2_), 3.52 (t, ^3^*J* = 4.9 Hz, 4H, CH_2_), 3.45 (t, ^3^*J* = 4.9 Hz, 4H, CH_2_), 2.43 (s, 3H, CH_3_) ppm; ^13^C NMR: (101 MHz, CDCl_3_) δ = 169.2 (C=O), 143.9 (CSO_2_), 136.2 (C_Ar_), 130.0 (CH_Ar_), 127.1 (CH_Ar_), 70.8 (CH_2_), 70.5 (OCH_2_), 69.2 (OCH_2_), 49.9 (NCH_2_), 38.7 (NCH_2_), 21.6 (CH_3_) ppm.

**4.6. 4-Tosyl-1,7,13-trioxa-4,10,16-triazacyclooctadecan**

1.1 g (2.5 mmol, 1 eq) 16-Tosyl-1,7,13-trioxa-4,10,16-triazacyclooctadecane-3,11-dione were dissolved in 10 mL anhydrous THF under argon atmosphere. Subsequently, 11 mL of a 1 M BH_3_-THF solution (11 mmol, 4.4 eq) was added dropwise. The reaction solution was stirred for 12 h at 50 °C. At the end of the reaction time, 2 mL of deionized water and 4 mL of 12 M HCl were added dropwise and the solution was stirred for another 2 h at room temperature. The solvent was then removed. The residue obtained was dissolved in 5 mL deionized water and washed three times with 20 mL chloroform each time. The aqueous phase was raised to a pH >11 with lithium hydroxide and the product was extracted four times with 20 mL chloroform each time; the solution was dried with magnesium sulfate and filtered. After removal of the solvent, the product was obtained in a yield of 94 % (899 mg, 2.2 mmol) as a colorless solid. R_f_ = 0.16 (EtOH: EA, 3:2); ^1^H NMR: (400 MHz, CDCl_3_) δ = 7.67 (d, ^3^*J* = 8.2 Hz, 2H, ArH), 7.26 (d, ^3^*J* = 8.2 Hz, 2H, ArH), 3.59-3.49 (m, 12H, CH_2_), 3.47 (t, ^3^*J* = 4.7 Hz, 4H, CH_2_), 2.73 (dt, ^3^*J* = 13.4 Hz, ^4^*J* = 4.7 Hz, 8H, CH_2_), 2.40 (s, 4H, CH_3_) ppm; ^13^C NMR: (101 MHz, CDCl_3_) δ = 143.2 (C_Ar_), 138.0 (C_Ar_), 129.8 (CH_Ar_), 127.0 (CH_Ar_), 70.8 (OCH_2_), 70.1 (OCH_2_), 67.0 (OCH_2_), 49.3 (NCH_2_), 49.1 (NCH_2_), 48.5 (NCH_2_), 21.6 (CH_3_) ppm.

**7,19-Ditosyl-4,10,16,22,27-pentaoxa-1,7,13,19-tetraazabicyclo[11.11.5]nonacosane-2,12-dione**

4-Tosyl-1,7,13-trioxa-4,10,16-triazacyclooctadecane (997 mg, 2.4 mmol, 1 eq) and Et_3_N (1.9 mL) were dissolved in anhydrous THF (30 mL) and filled into a syringe (V = 30 mL, Ø 2 cm). *N*-tosyl-2,8-dioxa-5-aza-1,9-nonanedicarboxylic chloride (977 mg, 2.4 mmol, 1 eq) was dissolved in anhydrous THF (30 mL) and filled into a second syringe (V = 30 mL, Ø 2 cm). Both solutions were added dropwise (4.6 mL/h) into anhydrous THF (240 mL) with help of a syringe pump under argon at ambient temperature and the resulting mixture was allowed to stir overnight. Afterwards, the solution was filtered, the solvent was removed and the crude product was purified via column chromatography (SNAP 25 cartridge, flow rate 50 mL/min; EA:EtOH, 0-20 % in 20 CV) to obtain compound **3** (797 mg, 45%) as a yellowish oil. R_f_ = 0.44 (ethyl acetate:EtOH + 0.1% Et_3_N = 2:3), ^1^H NMR: (400 MHz, CDCl_3_) δ = 7.69 (d, ^3^*J* = 7.9 Hz, 2H, H_Ar_), 7.33–7.27 (m, 4H, H_Ar_), 4.26 (s, 4H, CH_2_), 3.99–3.06 (m, 32H, CH_2_), 3.57 (t, ^3^*J* = 4.9 Hz, 4H, CH_2_), 3.52 (t, ^3^*J* = 4.9 Hz, 4H, CH_2_), 3.45 (t, ^3^*J* = 4.9 Hz, 4H, CH_2_), 2.43 (s, 3H, CH_3_) ppm; ^13^C NMR: (101 MHz, CDCl_3_) δ = 169.2 (C=O), 143.9 (C_Ar_), 136.2 (C_Ar_), 130.0 (CH_Ar_), 127.1 (CH_Ar_), 70.8 (CH_2_CO), 70.5 (CH_2_O), 49.9 (CH_2_N), 38.7 (CH_2_N), 21.6 (CH_3_) ppm.

**4,10,16,22,27-Pentaoxa-1,7,13,19-tetraazabicyclo[11.11.5]nonacosane (1)**

7,19-Ditosyl-4,10,16,22,27-pentaoxa-1,7,13,19-tetraazabicyclo[11.11.5]nonacosane-2,12-dione (408 mg, 0.54 mmol, 1 eq) was dissolved in 10 mL anhydrous THF. Lithium aluminum hydride (400 mg, 10 mmol, 19.5 eq) was added in portions to this solution and the resulting solution was stirred for 20 h at 60 °C. Afterwards, 1 mL deionized water and 1 mL 10 % LiOH solution was added. MgSO_4_ was then added and the solution was filtered. The solvent was removed and the residue was dissolved in 2 mL deionized water. The solution was acidified with 1 M HCl to a pH <2 and washed three times with 20 mL chloroform. The aqueous phase was then adjusted with LiOH to a pH >11 and the product was extracted five times with 20 mL chloroform. After removing the solvent, Cryptand **1** was obtained in a yield of 99 % (226 mg, 0.54 mmol) as a colorless oil. R_f_ = 0.82 (EtOH: EA = 3:2), ^1^H NMR: (400 MHz, CDCl_3_) δ = 3.65-3.50 (m, 20H, CH_2_), 2.87-2.67 (m, 20H, CH_2_) ppm; ^13^C NMR: (101 MHz, CDCl_3_) δ = 69.9 (OCH_2_), 69.1 (OCH_2_), 55.4 (NCH_2_), 54.7 (NCH_2_), 49.0 (NCH_2_) ppm; MS (ESI+): *m/z* = 419 [M+H]^+^.

**NMR spectra of compounds**

Cryptand **1**

Compound **4a**

Compound **5a**


Compound **7a**

Compound **8**

Compound **9**

Compound **10**

Compound **11**

**NMR Titrations**

Titration of ligand **8** with Lu^3+^

Titration of ligand **9** with Lu^3+^

Titration of ligand **10** with Lu^3+^

Titration of ligand **11** with Lu^3+^

Titration of ligand **8** with In^3+^

Titration of ligand **9** with In^3+^

Titration of ligand **10** with In^3+^

Titration of ligand **11** with In^3+^
